# Patient-Centered Medicine: A Necessary Condition for the Management of Functional Somatic Syndromes and Bodily Distress

**DOI:** 10.3389/fmed.2021.585495

**Published:** 2021-04-27

**Authors:** Pascal Cathébras

**Affiliations:** Department of Internal Medicine, Jean-Monnet University, Saint-Etienne, France

**Keywords:** patient-centered medicine, functional somatic syndromes, bodily distress, psychosomatic, dualism, reassurance

## Abstract

This paper argues that “functional,” “medically unexplained,” or “somatoform” symptoms and disorders necessarily require a patient-centered approach from the clinicians. In the first part, I address the multiple causes of the patients' suffering and I analyze the unease of the doctors faced with these disorders. I emphasize the iatrogenic role of medical investigations and the frequent failure in attempting to reassure the patients. I stress the difficulties in finding the right terms and concepts, despite overabundant nosological categories, to give a full account of psychosomatic complexity. Finally, I discuss the moral dimension attached to assigning a symptom, at times arbitrarily, to a psychogenic origin. The following part presents a brief reminder of the patient-centered approach (PCA) in medicine. In the last part, I aim to explain why and how patient-centered medicine should be applied in the context of functional disorders. First, because PCA focuses on the patients' experience of illness rather than the disease from the medical point of view, which is, indeed, absent. Second, because PCA is the only way to avoid sterile attribution conflicts. Last, because PCA allows doctors and patients to collaboratively create plausible and non-stigmatizing explanations for the symptoms, which paves the way toward effective management.

## Core Issues Posed by Functional Disorders

Symptoms and syndromes labeled as “functional,” “medically unexplained,” “psychosomatic,” “somatoform,” and so on are and will remain common, despite advances in medical science. Sometimes, they involve isolated symptoms, temporary or long-lasting; sometimes, they are relatively clearly defined (yet with labels that often change with a fragile nosology reflecting the controversy surrounding their causes and medical legitimacy) (1, 2); while at other times, they constitute idiosyncratic complaints that, at best, confuse doctors and, at worst, exasperate them. Though it is feasible to assume that their prevalence varies depending on how sophisticated the medical means used in explaining them, the fact that they are highly common and cause a considerable cost to health care systems cannot be denied (3, 4). Somatoform disorders [which are persistent medically unexplained symptoms (MUS) leading to impairment and distress] occur in ~6% of the population, 16% of primary care attenders, and up to 33% of patients in secondary care clinics (3). Specific functional somatic syndromes are also common, with prevalence estimates around 7% for irritable bowel syndrome, 2.5% for chronic fatigue syndrome, and 2–4% for fibromyalgia (3). Somatoform and functional disorders are associated with increased health care use and costs, and represent major reasons for sickness absence, disability benefits, and early retirement pensions in Europe and the US (3).

These functional conditions, symptoms, and syndromes pose many issues that may contribute to a frustrating and often iatrogenic doctor–patient relationship; among the issues are the following.

### Three-Fold Issues for Both Patients and Doctors

While telling a patient there is “nothing wrong” should be reassuring, in many cases, it is instead a cause of suffering for both patient and doctor. For the patient, the suffering due to symptoms (discomfort, pain, fatigue, and limits to daily life) is compounded by the uncertainty about their cause (and fear about their outcome), and the frustration induced by the lack of medical legitimacy. Doctors experience similar uncertainty regarding the nature of the patient's symptoms (and thus fear of misrecognizing a “medical” disorder), as well as difficulty of reassuring patients and powerlessness in front of refractory symptoms (4).

### The Fear of Diagnostic Errors and the Management of Uncertainty

Symptoms are labeled as “medically unexplained” when there is no proven organic cause, yet physicians always fear to leave a “medical” cause undetected. Doctors always bitterly regret having missed a physical diagnosis, yet curiously never deplore having contributed, through extensive workup and uncoordinated referral to medical specialists, to the genesis of refractory somatization (5, 6). Uncertainty has become more and more intolerable for doctors (7), which have long been affected by the “futile search for certainty” that pushes them to order and perform more investigations (8). Diagnostic errors (labeling as functional symptoms explained thereafter by an organic disease) are nevertheless rare and do not always have harmful consequences (9, 10). Moreover, unlike common assumptions, patients that present with functional disorders do not always “push” their doctors to prescribe supplementary tests, or at least not to the extent they assume (6, 11, 12).

### The Ambiguities of Reassurance and the Perverse Effects of Searching for Organic Causes

Most patients with functional disorders are worried, and doctors wish to reassure them. However, this crucial skill is far from simple and is not taught in medical schools (13). Besides, it is not always even appropriate, as the extent of disability and social consequences of some chronic functional disorders are sometimes much worse than those of organic diseases (4), as Michael Balint pointed out: “*In some cases, a physical illness represents, in fact, a more serious threat to the patient's well-being, but in others, the functional illness is definitely the greater danger*” (14).

Prescribing complementary tests expected to yield a negative result is generally used by doctors as a way of reassuring patients. However, this is a dangerous illusion (13, 15), since ordering test after test with the aim of “ruling out” an organic disease feeds the belief that “something has to be found,” reinforces anxiety while waiting for the results, and eventually increases the focus on symptoms while deceiving the patient's expectations for explanation when tests prove negative. In the worst-case scenario, complementary tests find abnormalities without significance (biological or radiological “incidentalomas” or false positives, especially common when the disease to be ruled out is unlikely), leading to more tests and consultations, further increasing uncertainty and anxiety, encouraging the patient to adopt a sick role, and eventually contributing to the chronicity of the condition (5, 6, 11, 12). Balint stated that “*It is implied. that the patient is not changed or influenced by the process of ‘elimination' (but) the patient's attitude to his illness is usually considerably changed during and by the series of physical examination*” (14).

### The Traps of Nosology

The terms used in discussing functional disorders are numerous and the nosology in this field is unsatisfactory. The term *functional* qualifies the subjective nature of a symptom as opposed to one of *organic* origin (caused by a lesion or relating to an established physiopathology). It is a rather neutral term (not necessarily involving psychogenesis) and the most easily accepted by patients (16). The term *medically unexplained* brings up this pervasive belief that the primary task of doctors is to rule out an organic cause for the presented symptoms, giving the impression that without such a cause, they do not fall under the responsibility of medicine. At the very worst, it could be taken as a denial of the symptoms and suffering, or by default as the assertion of their psychogenic nature. Moreover, considering a given symptom as fully “explicable” does not take away the arbitrariness of the decision. The term *somatization* means, according to authors, a psychopathological process of “converting” distress or more or less conscious psychological conflicts into physical symptoms; a form of “illness behavior” characterized by the “*tendency to experience and communicate somatic distress in response to psychosocial stress and to seek medical help for it*” (17); or simply the persistent presence of debilitating functional somatic symptoms (18). The last two definitions differ from the first in that they are not predicated on the symptoms being (solely) psychogenic. *Somatoform disorders* are a group of mental disorders introduced in the third edition of the Diagnostic and Statistical Manual of Disorders (DSM-III) that all featured somatic symptoms are with no physical explanation and health care seeking. The categorization and even the very idea of somatoform disorders were severely criticized by many researchers of the field (19), leading to a complete overhaul creating the 5th edition (DSM-5), which identified a separate diagnostic group labeled *somatic symptom disorders*. The “medically unexplained” nature of the symptoms is no longer mandatory, while psychological distress and abnormal illness behavior associated with physical symptoms have become a major criterion, and the category has also been widened to include factitious disorders and “psychological factors affecting other medical conditions,” which were previously found in other sections of the DSM. Unlike somatic symptom disorders, which are categories developed by psychiatrists, *functional somatic syndromes* (FSS) are diagnostic labels coined by non-psychiatric physicians that refer to syndromes without an organic disease explanation, structural changes, or established pathophysiology, for which each medical specialty has at least one example, such as fibromyalgia for rheumatology, irritable bowel syndrome for gastroenterology, chronic fatigue syndrome for internal medicine and infectious diseases, hyperventilation syndrome and non-cardiac chest pain for respiratory medicine and cardiology, and so on. These syndromes, which display overlapping clinical features, frequently occur in association, and thus singling any of them out as a specific entity could simply just be the result of medical specialization (20). Indeed, there is supporting evidence for shared risk factors, triggers, and perpetuating factors across these disorders, as well as for the efficacy of similar treatment approaches (21). In line with this “lumping” view, a new category called *bodily distress disorder* has been constructed to capture most of the “somatoform” and “functional” disorders under a single diagnostic category (22), and this diagnostic term has been chosen to replace the somatoform disorders in ICD-11.

Due to this overabundant nosology, a patient suffering from functional symptoms may receive several different diagnostic labels, such as one or more among the functional somatic syndromes as well as one or more somatoform disorder, not to mention the anxiety and mood disorder diagnoses frequently associated with them which may sometimes explain all or some of the symptoms. Depending on the diagnostic term used, the psychogenic nature of the symptoms is either explicitly affirmed or implicitly assumed, and the associated psychological distress was viewed either as a causal factor or as mere comorbidity. This ambiguous and complex nosology makes communication between different health care professionals complicated, while it is of vital importance that they collaborate closely in order to manage and treat patients. Furthermore, doctor–patient communication is also hampered, with any attempt at offering the clear information that every patient deserves becoming near impossible.

Recently, the EURONET-SOMA group proposed “*functional somatic disorder*” as an umbrella diagnostic term for the various conditions characterized by persistent and troublesome symptoms, to be situated in a neutral space within disease classifications, favoring neither somatic disease etiology nor mental disorder (23). A wide adoption by the medical and psychological communities of this new classification would, in my opinion, be a great progress toward the resolution of the pervasive dualism that impedes communication with patients and between health care professionals, understanding, and proper management of “medically unexplained” symptoms and bodily distress.

### The Moral Valence of Dualism and the Problem of Legitimacy

The Cartesian dualism of mind and body are deeply ingrained across Western society and can be considered as an epistemic pillar of biomedicine. Not only is a symptom considered as more “real” if caused by an organic disorder than if “unexplained,” but there is a moral judgment of responsibility, if not of blame, imposed on symptoms suspected as being of psychological origin. This moral weight of dualism explains that the persons suffering from physical symptoms that they feel they have no control over tend to attribute them to a medical condition that they could in no way be considered as responsible. They understandably prefer to be seen as victims, legitimately requiring the help of medicine, rather than suspected culprits. Sociological studies of people suffering from functional disorders have extensively documented their painful experiences of misunderstanding and suspicion from the biomedical establishment, which underestimates their suffering and the pain of their symptoms, only grudgingly according them the status of patients that they believe is theirs by right (24). For some sufferers, the quest for medical legitimacy appears to take priority over looking for means to alleviate symptoms. This has deleterious consequences: as Nortin Hadler coined it, how can you get well if you have to prove you are ill? (25).

Questioning the validity of some of the “myths” that underlie our understanding of MUS, using the most recent research available (26), should help in avoiding some of these traps of dualism. MUS are not always associated with depression and anxiety, while “organic” disorders are often complicated by psychological distress or common psychiatric disorders. MUS are often associated with physical conditions, and the underlying mechanisms are not fundamentally different from those of organic diseases, with biological and psychological factors always at work: the pathophysiology of the major functional somatic syndromes is a good example of how complex their interactions can be (22).

The issues addressed in this chapter help pave the way toward a more constructive approach to managing functional disorders. Firstly, to address the multiple causes of a patient's suffering and to analyze the unease experienced by health care providers when faced with these disorders; secondly, to measure the iatrogenic role of medical investigations and to understand why most attempts to reassure the patient do not work; thirdly, to assess how difficult it is to find the right terms and concepts to give full account of psychosomatic complexity; and finally, to be conscious of the moral dimension attached to assigning a symptom, at times arbitrarily, to a psychogenic origin. This brought about an attempt of conceiving the problem from the patient's point of view, in order to help him/her get through it.

## A Medical Approach Focused on the Patient/Person

The tensions between medicine focused on the disease/doctor and those centered on the patient/person are nothing new, and no explanation offered by biological sciences or advances in treatments of evidence-based medicine has changed anything. Take, for example, the aphorism variously attributed to Hippocrates, William Osler, and others, stating that it is “more important to know what sort of person has a disease than to know what sort of disease a person has” to be a good doctor. Some link this concept with the concern expressed by doctors throughout the ages about the risk of medicine becoming too scientific, turning doctors away from their humanist values. The idea of a doctor viewing and understanding a patient as a “whole” person is also central to the studies and life's work of Michaël and Enid Balint, the latter of whom is credited with being the first to use the term “patient-centered medicine” in the literature (27): “*There is another way of medical thinking which we call ‘patient-centered medicine.' Here, in addition to trying to discover a localizable illness, the doctor has to examine the whole person [.]. This should include everything that the doctor knows and understands about his patient; the patient, in fact, has to be understood as a unique human being*.” The patient-centered approach (PCA) is connected to the landmark “bio-psycho-social” theoretical model of George Engel (28, 29) and the operationalization of competing terms *illness, disease*, and *sickness* by Harvard psychiatrists and anthropologists (30) (to be discussed later), both of which appeared in the 70s. Yet, it was not until the 80s that the concept finally gained worldwide recognition, promoted by the family medicine department of Western University, Ontario (31). In its current form, the concept aligns with criticisms of the “paternalist” carer–patient relationship model and promoters of a “participatory” model that focuses on patient autonomy. It also echoes the distinction between cure and care. Finally, this approach has found its place in the “narrative medicine” movement (32) and modern conceptions of patient education and counseling. Whatever its form, it establishes that patients possess knowledge on their “illness,” that their psychological and behavioral reactions are impacted by their representations, and that they own resources that we must help them access and use to improve their health and decrease the suffering and social consequences of by their health condition.

PCA aims to combine traditional medical activities such as diagnosis and treatment (based, as far as is possible, on factual evidence) with the explicit understanding of what patients personally experience, including what they perceive and how they explain what is wrong, their emotions and feelings regarding their condition, especially their fears, the impact of their health issues on their day-to-day activities and functions, and their expectations of what should be done. According to its promoters (31), the implementation of PCA comprises six interactive components that are formulated as orders for doctors to follow (List 1). Recent reformulations of the basic elements of patient-centered care have been offered (List 2) (33). Many academic programs have been created to train doctors in PCA (34), especially in terms of interviewing and communication techniques (35). However, although health care organizations currently strive to improve health care system performance through the implementation of person-centered care, PCA is often “more preached than practiced,” and despite a large consensus on the values carried by the concept, actual implementation of PCA at the health care delivery level remains a challenge (36, 37).

**List 1:** The six components of the patient-centered process (31).

1. Exploring both the disease and the illness experience a) differential diagnosis b) dimensions of illness (ideas, feelings, expectations, and effects on function) 2. Understanding the whole person a) the person (life history and developmental issues) b) the context (family and significant others, physical environment) 3. Finding common ground regarding management a) on the problems and priorities b) on the goals of treatment c) on the respective roles of doctor and patient 4. Incorporating prevention and health promotion 5. Enhancing the patient–doctor relationship a) sharing power b) building a caring and healing relationship c) promoting self-awareness d) recognizing transference and countertransference 6. Being realistic, taking into account: a) time b) resources c) team building

**List 2:** Elements of patient-centered care (33).

1. The health care system's mission, vision, values, leadership, and quality-improvement drivers are aligned to patient-centered goals. 2. Care is collaborative, coordinated, and accessible. 3. Care focuses on physical comfort as well as emotional well-being. 4. Patient and family preferences, values, cultural traditions, and socioeconomic conditions are respected. 5. Patients and their families are an expected part of the care team and play a role in decisions at the patient and system level. 6. The presence of family members in the health care setting is encouraged and facilitated. 7. Information is shared fully and in a timely manner so that patients and their family members can make informed decisions.

The benefits of PCA are potentially great, though the scientific evidence of these positive effects is weak (in part due to the difficulties in creating research contexts where the many ingredients of PCA can be operationalized, but also due to a lack of modeling of the mechanisms linking doctor–patient communication with patient health) (38–40). PCA has been shown to allow obtaining deeper and more pertinent information (including for establishing a biomedical diagnosis), to generate greater patient satisfaction, to achieve better compliance, to provide stronger reassurance, and to enable more effective therapeutic relationships (31, 40). Studies on patient perception have confirmed these expectations (41). Importantly, doctors who practice PCA report higher satisfaction themselves, and despite what many think, patient-centered consultations have been found to be no longer than traditional ones and could even save time (31, 35, 42). Patient-centered care has been associated with decreased use of diagnostic tests and referrals and decreased health care utilization (43, 44). Patient-centered care may thus improve resource allocation and reduce expenses throughout the continuum of care (33). Finally, it is likely that PCA helps to some extent to forewarn against lawsuits (45).

PCA has been criticized for the risk of diverting the physicians' attention from diagnostic issues, or for being difficult to implement due to time constraints in the consultation room. However, the main problems with PCA may well be practice gaps due to the widespread context of biomedically health care, the lack of emphasis on PCA in medical education, and the lack of supportive environment and financial incentives for practicing physicians (37).

The concept of patient-centered care extends for some authors to the customization of treatment based on individual biological characteristics, known as “personalized medicine,” broadly conceived as the use of genomics, proteomics, and other recent technologies to provide decisions with regard to prediction, diagnosis, and treatment of disease (33). Such an approach may be relevant to the treatment of functional disorders in the tradition of Engel's biopsychosocial model. Notably, systems thinking (46) is propelling research on persistent somatic symptoms, whether “medically unexplained” or not, beyond mind–body dualism and toward the integration of psychological, biological, and social contextual factors, using modern technology to link the subjective experience of somatic symptoms with objective measures of biological processes such as autonomic imbalance (47). However, some authors have argued that systems medicine remains insufficient in bridging the gap with humanistic medicine built on the concept of “patient as a person.” They consider that, being at least for the moment unable to account for meaning, value, and symbolic interaction, systems medicine cannot be fully integrative, holistic, and patient-centered in a humanistic sense (48). The question of whether PCA and personalized medicine are irreconcilable concepts or may be combined at theoretical and pragmatic levels is a subject of philosophical inquiry (49).

## Why Patient-Centered Medicine Should be Applied in the Context of Functional Disorders

### PCA Focuses on the Patient's Experience of Illness Rather Than the Disease From the Medical Point of View (Which Is, in Such Cases, Absent)

To begin with, we need to go back to the dichotomy between *disease* and *illness*, set into motion by medical anthropologists in the 70s and 80s (30, 50) building on René Leriche's concept of the “doctor's illness” as opposed to the “patient's illness.” The term *disease* represents the illness as an objectifiable biological reality. In this approach, the focus is on what is objective and, where possible, quantifiable; the disease is a “thing on its own” (ontological perspective) and medical inquiry aims to sort through the patient's experiences retaining as pertinent only their symptoms (which thus become signs) and complaints that can be linked to an anatomical or biochemical abnormality, or a well-defined pathological entity in the case of psychiatry. For the doctor, the ultimate “reality” is that of the disease: the social and cultural context of the sick persons, their emotions and personality, and the meaning ascribed to their ailments are contingent. The term *illness*, on the other hand, refers to the experience of disease, a fundamentally subjective and idiosyncratic reality that is also impacted by the cultural and social context, communicated to other people, covering the perception of trouble, the emotions that accompany it, and the lay interpretation of symptoms. This experience is most often created through interaction with friends or family, and more or less explicitly involves the patient's pervasive questioning about the meaning of the disease as a personal misfortune, along the lines of “do I deserve this?” or, in other cultural contexts, “who wishes harm to me?” (51). Finally, the term *sickness* aims to more comprehensively cover the multiple social dimensions of what is already suggested in the term *illness*: how the definitions of health and sickness vary from person to person, culture to culture, and even across different social classes; the existence of culturally recognizable and socially acceptable forms of being sick (idioms of distress); and the status of sickness requires a social legitimation that is obtained through conforming to standards governing illness behavior. In many societies, and particularly our own, the sick role can actually be seen as a deviant yet not sanctioned behavior, instead prompting help and compassion, as long as the disease can be blamed on an agent located out of the person's control (52).

These concepts show us just how far the disease-centered approach becomes untenable as soon as functional or “medically unexplained” symptoms are in play, since this means treating *illnesses* without *disease*. Then, the only pragmatic response to these symptoms is to address the illness experience of a specific person. On the other hand, doctors cannot overlook that they play a key role in our Western societies in the social legitimization of an ill person's status by establishing a diagnosis and prescribing sick leave, among other responsibilities. When faced with someone presenting functional symptoms, experienced doctors often quickly realize that an actual disease will not be found, but they can still choose to focus on the disease rather than on the patient, because there lies their field of expertise and skill, but also for fear of hastily taking the risk of delegitimizing the patient's complaint. Balint coined that “*diseases are arranged in a kind of ranking order roughly corresponding to the seriousness of the anatomical changes which can be demonstrated or assumed in them. Unfortunately, not only are the diseases given this kind of rank, but also the patients, so to speak, are attached to them. Patients whose complaints may be traced back to demonstrable or assumable anatomical or physiological changes are rank higher, and neurotics are in a way the dregs left over after everything else has been drawn off. Thus, it is understandable that every doctor, when confronted with a new patient, tries to give a good rank and will relegate the patient to the class of neurotics only if he cannot find any justification to give a respectable status*.” (14). This game of trickery helps to construct somatization, especially when a doctor proves incapable of seizing on the clues a patient offers him (6). Somatization can actually be fundamentally conceived as a dispute over attribution (“it's in my body” vs. “it's in his head”), yet empirical studies have shown that in a way this conflict may be overplayed by both parties (53), with patients often offering an opening for a psychosocial explanation for their symptoms (as long as that is not meant as their exclusive cause), and above all wishing for the doctor to be interested in their symptoms and not to immediately normalize them (6, 53).

### PCA Enables Attribution Conflicts to Be Explicitly Discussed and at Best Avoided, and for Doctors to Create With the Patient Plausible and Non-stigmatizing Explanations for the Symptoms

Combining the benefits of PCA contribution with a bio-psycho-social approach for functional disorders (54) requires certain useful assertions to be retained (List 3). It is not about abandoning the biomedical approach focused on research of identifiable organic diseases and evidence-based treatment, but rather to explore concurrently and simultaneously the “patient's agenda,” meaning their background and life context, their representations, fears, expectations, and emotions. There would be less stigmatization of functional disorders if the exploration of the psychosocial aspects of suffering was not reserved solely for patients whose symptoms resist medical explanation (54).

**List 3:** Principles of a biopsychosocial approach to unexplained somatic symptoms and functional disorders (54).

1. All significant illnesses affect patients on multiple levels (cellular, organismic, interpersonal, societal). 2. Somatic symptoms correlate poorly with pathological findings. 3. The emotions are embodied. 4. Somatic expression of psychological distress is a normal and universal phenomenon. 5. Language of medical discourse shapes patients' experience of illness as well as physicians' understanding of patient. 6. Stigmatization and blame further reinforce symptoms and conflict. 7. Many somatically distressed patients are chronically ill, requiring secondary prevention and functional assessment. 8. Treatment should be oriented toward care as well as cure and cause.

What is most important is to come to an agreement on how to call the problem (2): a name that would make sense both to the doctors and patients, which should lead toward normalizing symptoms rather than psychologizing them, thus avoiding stigmatization, and which would communicate plausible, understandable, and personalized explanations of the mechanisms underpinning the disorder. The term chosen should also convey the complexity [distinguishing, for example, between predisposing, triggering, and maintaining/aggravating factors of the symptoms (21)], as well as being congruent with the experience of the patient, while allowing to alleviate some suffering. This, of course, is a difficult task. Patients tend to resist psychosomatic attributions unilaterally attributed by doctors (55). A diagnosis of “somatoform disorder” has no useful meaning for most patients, and diagnoses of functional somatic syndromes are no more easier to understand (56), except for functional disorders based on unorthodox or controversial attributions (multiple chemical sensitivity, electromagnetic hypersensitivity, chronic Lyme disease, and so on), which evidently offer a “cause” and sometimes a “meaning” to the symptoms, but also tend to isolate the “victims” and often determine hostile patient–doctor relationships (57). In no event does a diagnosis of fibromyalgia, for example, dispense the doctor from searching for physical or psychiatric differential diagnoses, from compiling the list of the multiple contributions to the disorder (somatic, psychological, behavioral, or other), or to carefully explore the representations of the “disease” (including the representations of the label chosen: does this name convey the meaning of an “imaginary illness?” of a mental illness? of a “cover-up” diagnosis to hide the doctor's ignorance? a prognosis of incurability or, conversely, of benignity?). Nevertheless, the doctor should also attempt to create, with the patient's participation, an explanatory model that is relatively coherent and includes personal details such as, for example, a background of childhood abuse as a *vulnerability factor*; an acute infection as a *triggering factor*; and the core mechanisms of central sensitization of pain, physical deconditioning due to prolonged rest, poor sleep quality, over-focusing on the symptoms induced by the fear of severe illness and complementary investigations, anger caused by a lack of recognition, and insulting attitudes of certain physicians as perpetuating factors, which create ever-worsening vicious circles. We can therefore say that one name is not enough; in fact, being simultaneously “precise and wrong,” it could even be dangerous (2). The causality of functional symptoms is, by necessity, made up of many factors and varies from person to person. By exploring the experience and representations of the patient, the doctor can in his turn put forward his own descriptive or mechanistic metaphors, engaged with the patients in creating an interpretation of their disorder that can itself have a therapeutic effect (4, 16, 53, 54, 56, 58, 59).

How could PCA might be a solution to the core issues posed by functional distress listed in the first part of this article? I argue that PCA offers the opportunity of *explicitly* discussing with the patient these problematic issues, taking account of his/her point of view. As examples, about the issues of doctors' and patients' discomfort with unexplained symptoms and management of uncertainty (points 1.1 and 1.2), the doctor may acknowledge that he/she is at unease with not having a simple explanation for the symptoms and that he/she shares with the patient (in part) the fear of missing a rare or atypical medical disorder. He/she can address explicitly the issue of the suffering experienced by the patient due to the lack of explication/medical legitimacy for his/her symptoms. Does the patient feel he/she has to “prove” that he/she is genuinely ill? Concerning the issue of reassurance and complementary medical workout (point 1.3), the doctor should explicitly enquire about the patient's experience and understanding of the process of “ruling out” organic causes for the symptoms. Does the patient feel actually reassured by negative findings, or does he/she feel that this was just not the right test or the right specialist? Has his/her anxiety been soothed or aggravated by the medical investigations? With regard to the traps of nosology (point 1.4), the problems with the words used to give account of the situation can also be explicitly addressed with the patient (see above). Are the diagnoses of “somatoform disorder,” “fibromyalgia,” “bodily distress disorder,” and so on, understandable? Which of these terms are felt as insulting? Does the patient feel that they mean that “it is all in the head?” Finally (point 1.5), the doctor should also address explicitly the issue of dualism and legitimacy of symptoms with the patient. How does the patient view mind–body relationships? Does accepting a “psychosomatic” explanation mean that the patient is “responsible” for his/her symptoms? Examples of wording that may prove useful can be found in the Appendix.

### PCA May Be the First Step Toward Psychotherapeutic Approaches to Functional Disorders

Although the scope of this paper is primarily a medical one, with emphasis on doctor–patient communication in primary and specialized (non-psychiatric) care, it must be stressed that evidence-based psychological treatments of functional disorders exist (21, 60, 61). However, in many countries such as France, functional somatic disorders remain under recognized and undertreated, the clinical reality being often characterized by unstructured use of specialized somatic care, resulting in high costs (62). Barriers to diagnosis and treatment of functional somatic syndromes and bodily distress in primary care are numerous (63), and we have stressed only some of them. Furthermore, in some countries, many psychiatrists and psychologists feel uncomfortable with somatic symptoms and syndromes, may be unwilling to take care of patients often considered as “difficult” and frustrating, or are unaware of published guidelines and specific psychotherapeutic techniques, while specialized treatment centers for patients with FFS are rare or inexistent. Then, one of the major challenges to improve the management of functional somatic syndromes is to get patients into treatment.

PCA can help overcome barriers to psychological treatment for functional somatic symptoms in several ways. PCA requires a deep enquiry on patients' attributions and expectations, which is the starting point for cognitive restructuring and “reattribution” techniques used in cognitive behavioral therapies (CBTs) for somatoform disorders. Practicing PCA means being aware of patients' beliefs and expectations and of their willingness to offer or accept psychosomatic explanation, based on their previous experiences with health care providers, for instance. PCA also allows the inclusion of patients' idioms in the discussion about illness explanations (63), and the crafting of shared metaphors may help to engage them in treatment. Beyond beliefs and attributions, PCA explicitly addresses the patients' emotional correlates of, and response to, physical symptoms, thus opening the door to emotion regulation training as an enrichment of CBT for MUS, whose efficacy remains overall moderate (64).

## Summary and Conclusions

Although PCA is nothing new (the 60-year-old seminal work of Michael Balint having been quoted on purpose), this paper argues that its actual practice in clinical encounters with patients is a necessary condition for the management of functional symptoms and bodily distress. Its focus on *illness*, as the lived experience of symptoms of an individual patient and the idiosyncratic fears, expectations and feelings attached to them, allows the shift from the traditional *disease* perspective of biomedicine, which invariably leads to unhelpful conflicts of attribution, uncertainty-driven anxiety, distrust and frustration on both sides, as far as organic disorders have been reasonably ruled out or properly treated. PCA allows a therapeutic alliance to occur when the sociomoral issues of responsibility and blame are *explicitly* addressed and discussed with the patient (and at times with his/her family or significant others). When listened to carefully, most “somatizing” patients offer metaphors and sketches of explanations for their symptoms, on which doctors can help construct a shared model of psychosomatic entanglement contributing to the genesis of symptoms as predisposing, triggering, or perpetuating factors.

Functional symptoms and syndromes and “medically unexplained” or “psychosomatic” problems remain a blind spot of medicine. In a world of biomedically oriented medical education, a reminder of the person-centered approach seems at time mandatory. Of course, PCA should be learned as a set of clinical skill and not only as a theoretical framework. Much remains to be done within graduate and post-graduate medical education to effectively implement PCA in the management of bodily distress, an overlooked public health problem. However, this might not be enough. Since crucial moral issues are at stake with such conditions, medical education (especially within that field) should move, beyond competencies, to the development of a practical wisdom (*phronesis*), which link the knowledge and skills of biomedical and clinical science to moral orientations that addresses human interests in the practice of medicine (65).

## Data Availability Statement

The original contributions presented in the study are included in the article/Supplementary Material, further inquiries can be directed to the corresponding author.

## Author Contributions

The author confirms being the sole contributor of this work and has approved it for publication.

## Conflict of Interest

The author declares that the research was conducted in the absence of any commercial or financial relationships that could be construed as a potential conflict of interest.
