# Supplementary material for: Patient-Centered Medicine: A Necessary Condition for the Management of Functional Somatic Syndromes and Bodily Distress
Source: Front Med (Lausanne). 2021 Apr 27;8:585495. doi: 10.3389/fmed.2021.585495 (PMC8110699; doi:10.3389/fmed.2021.585495)
Supplement: Supplementary file 1 [file Data_Sheet_1.docx]

**Appendix: examples of wording**

1. Sharing uncertainty with patients, and finding common ground about further investigations to rule out an organic disease:

“*Your symptoms of dysesthesia and muscle weakness may suggest a neurological disorder such as MS, and I noticed and understood your fears of this disease. I wouldn't like myself to miss out such a disease. However, I have done a complete neurological examination which proved absolutely normal. I then would say that MS is your case very unlikely, but it can't be completely ruled out, and we have to see how things go on. I am not sure that prescribing a brain MRI would help at the moment. But I wish to examine you again in one or two months: if there are new symptoms despite the treatment for anxiety, and if I find any abnormality in the clinical examination, we could do the MRI and even think about a referral*

*to the neurologist. What do you feel about that?*”.

Should we share uncertainty with the patient, in which contexts, and how? The answer is not easy. Confidence should first be gained from the patient (age and professional experience of the practitioner are not the major factors if they are not associated with empathy). A careful physical examination, that may be reiterated, is a necessary condition and a powerful, but at times forgotten, tool for patient reassurance [1]. Whatever the choices made at the end (for example ordering tests or not), the doctor must show that he/she will fully assume the decision (ideally a decision made consensually with the patient) and accept to revise it. However, some patients may become too anxious when sharing uncertainty, and at times adopting a paternalist attitude of reassurance (which is easier if you are an older doctor) may be more appropriate.

2. Talking about the potential perverse effects of searching for organic disease:

“*We should discuss about ordering MRI in your case. What will we do if MRI proves normal? Do you think you will then feel reassured, or shall we have to do more tests or seek a specialist’s opinion? And what do you think we will have to do if MRI shows minor abnormalities that cannot explain your symptoms? This is not rare to find minor abnormalities on brain MRI in healthy people. Could such findings aggravate your anxiety? Do you think it is worth taking that risk?*”

Diagnostic testing undertaken to convince patients that their symptoms are benign can have unintended negative consequences, as many patients are not reassured by negative findings, and merely prescribing diagnostic testing may inadvertently validate and reinforce convictions that the symptoms are serious. Iatrogenesis is increased when test findings are inconclusive and is especially high if further testing is necessary to investigate a false positive result [2-3].

3. Avoiding the traps of nosology

“*How should your condition be labeled? I think the best way is to call it a “functional neurological disorder”. That means that it does not arise from damages of the brain or nerves, but that they do not function properly. Have your symptoms received other names from the doctors you saw earlier? How did you feel about the terms used? Did you find some labels offensive (e.g. “medically unexplained”, “psychosomatic”, etc.)? Were you told that “it was all in your head”? The term “functional”, in my mind, is not an euphemism for “psychogenic”, and I am certain that you are not imagining your symptoms. Functional neurological symptoms like yours are quite common, and because there is no damage in the nervous system, the patients I see get better most of the time. Functional symptoms are not due to psychological disorders, but stress and anxiety often make them worse. Functional symptoms usually result from many causes, some being biological, other psychological or social. They differ from person to person, so we should try to identify together, in your case, the more salient etiological factors, and what to do to get better. Do you feel comfortable with the term “functional”? Would you like to further explore the potential causes of your symptoms?*”

As Stone *et al*. pointed in their article on the management of functional neurological symptoms [4], the word “functional” replaces an erroneous physical versus psychological debate, allowing for a more productive functional/reversible versus structural/irreversible dichotomy; provides a rationale for any treatment designed to improve the functioning of the nervous system and then allows the use of both physical and psychological strategies; and avoids offence and thus can be used transparently with the patient. The term “functional somatic disorder”, which is neither purely somatic nor purely mental, reflects both the etiological evidence of the complex interactions between brain and body and the need to resolve the historical split between somatic and mental disorders, and has been favored by prominent authors in psychosomatic research [5].

**References:**

[1] Sapira JD (1972). Reassurance therapy: what to say to symptomatic patients with benign diseases. Ann Intern Med, 77:603-604.

[2] Howard LM, Wessely S (1996). Reappraising reassurance: the role of investigations. J Psychosom Res, 41:307-311.

[3] Penzien DB, Rains JC (2007). Reassuring patients about normal test results. BMJ, 334:325.

[4] Stone J, Carson A, Sharpe M (2005). Functional symptoms in neurology: management. J Neurol Neurosurg Psychiatry, 76(suppl. 1):i13-i21.

[5] Burton C, Fink P, Henningsen P, Löwe B, Rief W (2020). Functional somatic disorders: discussion paper for a new common classification for research and clinical use. BMC Med 18:34.
